# Organizational structure and the periphery of the gene regulatory network in B-cell lymphoma

**DOI:** 10.1186/1752-0509-6-38

**Published:** 2012-05-14

**Authors:** Ricardo de Matos Simoes, Shailesh Tripathi, Frank Emmert-Streib

**Affiliations:** 1 Computational Biology and Machine Learning Lab, Center for Cancer Research and Cell Biology, School of Medicine, Dentistry and Biomedical Sciences, Queen’s University Belfast, Belfast, UK

**Keywords:** B-cell lymphoma, Gene expression data, Gene regulatory network, Statistical network inference

## Abstract

**Background:**

The physical periphery of a biological cell is mainly described by signaling pathways which are triggered by transmembrane proteins and receptors that are sentinels to control the whole gene regulatory network of a cell. However, our current knowledge about the gene regulatory mechanisms that are governed by extracellular signals is severely limited.

**Results:**

The purpose of this paper is three fold. First, we infer a gene regulatory network from a large-scale B-cell lymphoma expression data set using the C3NET algorithm. Second, we provide a functional and structural analysis of the largest connected component of this network, revealing that this network component corresponds to the peripheral region of a cell. Third, we analyze the hierarchical organization of network components of the whole inferred B-cell gene regulatory network by introducing a new approach which exploits the variability within the data as well as the inferential characteristics of C3NET. As a result, we find a functional bisection of the network corresponding to different cellular components.

**Conclusions:**

Overall, our study allows to highlight the peripheral gene regulatory network of B-cells and shows that it is centered around hub transmembrane proteins located at the physical periphery of the cell. In addition, we identify a variety of novel pathological transmembrane proteins such as ion channel complexes and signaling receptors in B-cell lymphoma.

## Background

The inference of gene regulatory networks from gene expression data is crucial for enhancing our understanding about relations between genes [1-3]. In general, a gene network describes a map of direct physical (biochemical) interactions among genes, gene products or metabolites that occur in the living cell [4,5] and, hence, enable a systems biology approach [6-8]. It has been demonstrated that gene regulatory networks, as a specific type thereof, can be indirectly inferred from steady state gene expression data, which are measured under different conditions either in individual tissues or cell types [9-11].

In general, it is believed that the gene regulatory network is governed by major hub genes like transcription factors that directly bind specific DNA segments in the nucleus and activate or repress the expression of other genes [1,12]. Further, it has been proposed that the genes in cellular networks are organized by a hierarchical and modular structure. This assumption has been studied, e.g., for metabolic networks [13]. A hierarchical modularity implies functional community structures of interconnected layers in the network with a potentially heterogeneous modularity structure. For example, for the protein network of *E. coli* it has been demonstrated that the center of the network has a higher modularity than the periphery of the network [14]

**Figure 1:**
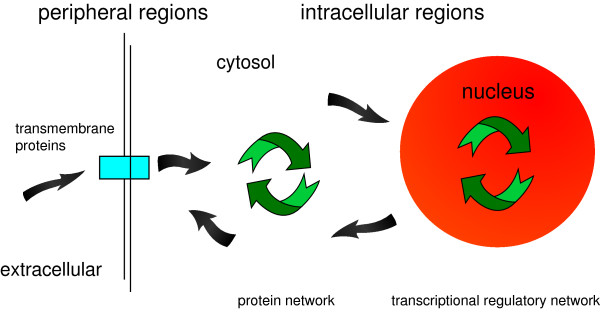
The gene regulatory network is composed of the transcriptional regulatory network, protein network and a signaling network spanning the whole cell.

In the following, we consider the periphery of a network to be given by leaf genes or linearly connected genes, while the central regions are complex, composed of genes with a high node degree. In [15] the functional modularity of different layers in the yeast and the *E. coli* protein network was observed to be governed mainly by a central and a peripheral layer, connected by an intermediate layer exhibiting a reduced modularity. The central layers of these networks were described to be highly enriched by genes that are located in the nucleus for regulating, e.g., the cell cycle, while the periphery is governed by metabolic, transport systems and cell communication processes. These results are consistent with the simplified view that the physical periphery of a cell produces signaling cascades that are induced by extracellular signals that are detected by transmembrane protein receptors. In turn, this leads to a transduction and amplification of extrinsic and intrinsic signaling cascades through the cytoplasm to the nucleus culminating in the regulation of gene expression. For an intuitive visualization of these intricate processes see Figure 1.

The inference of gene interactions in a gene regulatory network from gene expression data is often discussed in connection with the nuclear transcriptional regulatory network [1,16,17]. In the simplified transcription factor vs target gene model, a transcription factor affects directly the gene expression of the mRNA of a target gene. This may give the impression that gene interactions inferred from expression data need to be interpreted in the context of transcription regulation. For this reason, inferred networks from gene expression data are frequently equated with the transcriptional regulatory network. However, this is not justified because expression data convey only information about the dynamic state of genes correspondingly their mRNAs and, hence, do not provide direct information about any type of biochemical binding, including transcription regulation, at all. Instead, inferred interactions from expression data are not limited to transcription regulation, but can also include protein-protein interactions [18]. To emphasize this, we use the terminology *gene regulatory network* for a network that is inferred from gene expression data to point out that this is not necessarily a transcription regulatory network but a mixture of this and a protein-protein network [19].

The major purpose of this paper is to infer a gene regulatory network from a large-scale B-cell lymphoma gene expression data set, and to investigate its structural and biological organization. Immature B-cell lymphocytes are cells from the bone marrow that play an important role in the adaptive immune system. When B-cells are activated by an antigen they differentiate to memory B-cells, to antibody secreting plasma B-cells or proliferate intermediately to germinal centers (centroblasts and centrocytes) [20]. B-cells are one of the most interesting cell types for the study of mammalian signaling and cell differentiation processes due to their unique physiological properties governing the adaptive immune system. Malignancy of the different B-cell lymphocyte types leads to a variety of lymphoma and leukemia disease phenotypes such as *B-cell chronic lymphocytic leukemia* (BCLL, germinal center), *Burkitt lymphoma* (BL, germinal center), *Diffuse large B-cell lymphoma* (DLBCL, germinal center), *Follicular lymphoma* (FL, germinal center), *Hairy cell leukemia* (HCL, memory B-cells), *Mantle cell lymphoma* (MCL, immature B-cells) and *Multiple myeloma* (MM, plasma cells). For our analysis, we use the microarray data set from [21] which contains samples from the germinal centers of lymphoma patients and experimental transformed germinal center cell types.

In a previous study, it has been found that the C3NET inference algorithm has a considerably higher *true positive* (TP) rate for leaf edges of genes in a network that are sparsely connected [18]. For this reason we hypothesize that this method has characteristics which are very beneficial for the inference of peripheral regions of the gene regulatory network of B-cells. Due to the fact that B-cells are highly receptive to external stimuli, as described above, knowledge of these interactions seems viable for gaining a deeper functional understanding of the intricate differentiation processes.

In order to analyze the structural organization of B-cell lymphoma, we infer a gene regulatory network by using C3NET in combination with an ensemble approach. This means, instead of applying the inference method to one data set, we are applying it to a bootstrap [22] ensemble of data sets. This allows not only to assess local network-based measures down to the level of individual edges [23,24] but also to obtain an *average* network structure which is amenable for a hierarchical analysis, as we will show in this article.

There are several large-scale B-cell lymphoma related gene expression data sets available of germinal center tumor samples from *Diffuse large B-cell lymphoma* (DLBCL), *Follicular lymphoma* (FL) and *Burkitt lymphoma* (BL) [25-29]. In this paper, we study the gene regulatory network from B-cell lymphoma by using the data set in [21]. For an independent validation of our results we study in addition two Diffuse large B-cell lymphoma data set described in [25,27].

**Figure 2:**
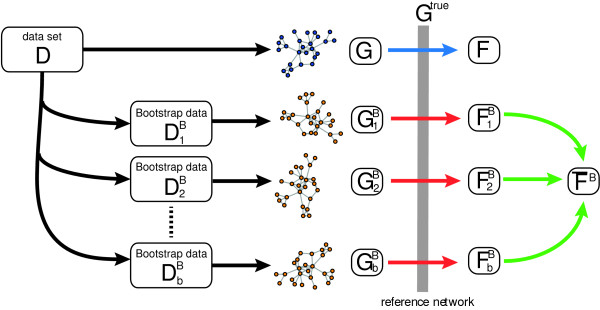
**Illustration of our simulation set-up to generate bootstrap data sets.** Using the true underlying network *G*^*true*^ as reference, we estimate F-scores for each of the inferred networks. The colors of the arrows correspond to the boxplots in Figure 5. Data set *D*_*k*_^*B*^ represents one data set of the whole ensemble.

To demonstrate the validity of our bootstrap approach, we are using simulations comparing results from a bootstrap ensemble with an ensemble of independently generated data. For a principle overview of the generation of the bootstrap data, see Figure 2. In this figure, the data set $D_{k}^{B}$ refers to the k-th data set from the bootstrap ensemble.

**Figure 5:**
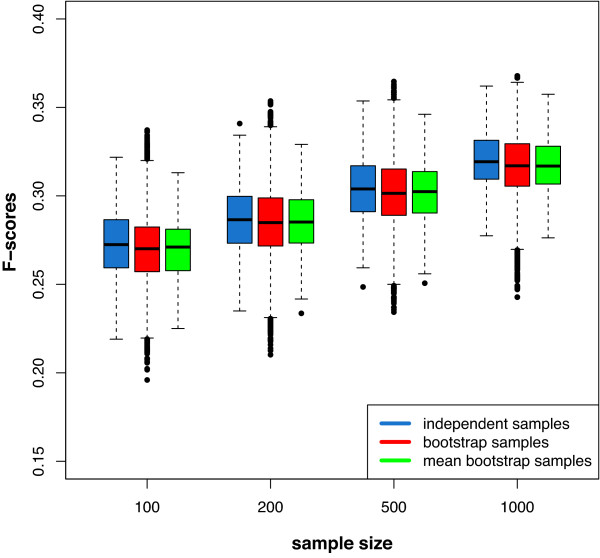
**Comparing the F-scores for inferring a scale-free network.** Blue: 300 independently simulated gene expression data sets. Red: BE 1. Green: BE 2. The data sets were generated with sample sizes ranging from 100, 200, 500 to 1000 samples

In this paper, we infer the peripheral region of the gene regulatory network inferred from a large-scale B-cell lymphoma gene expression data set by using the C3NET algorithm. We provide a functional and a structural analysis of the largest connected component for this network. Further, we analyze the hierarchical organization of the network components of the B-cell gene regulatory network as revealed by the bootstrap approach.

## Methods

In the following section we present the methods and the data used for our analysis.

### Simulated Gene Expression Data

We simulate gene expression data sets for a variety of different network structures by using SynTReN and GeNGe [30,31]. For each network type, we generate 300 data sets with a sample size of 100, 200, 500 and 1000. Further, for each of these data sets, a bootstrap ensemble of size *b* = 100 was generated by sampling with replacement.

**Figure 4:**
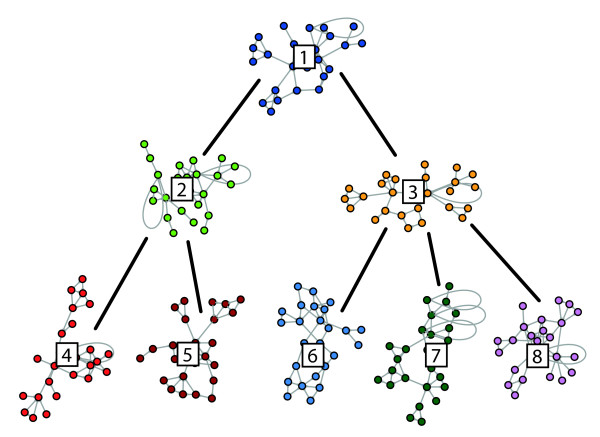
**Reference hierarchical network with 8 weakly connected MTM network modules.** Each module consists of 25 genes. The modules are connected by 0, 3, 5, 10 and 15 edges, as indicated by the black, thick edges between the modules.

In addition, we generate simulated gene expression data sets for a network consisting of 8 network modules, which are organized in a hierarchical manner; see Figure 4 for a visualization. Each network module is generated using a *Modular Topology Model* (MTM) network model, each with a size of 25 genes. A MTM network has properties such as a scale-free degree distribution, high clustering coefficients and short path lengths as observed in real biological networks [32,33]. We construct 5 different networks by weakly connecting the 8 individual modules with a different number of connections. Specifically, the individual network modules are connected by 0, 3, 5, 10 and 15 edges, resulting in a total of 5 networks, each consisting of 200 genes. For each of the 5 networks, we generate independently 100 gene expression data sets with sample size 500 by using netsim [32]. Netsim generates time-series data. In order to obtain steady state expression data each sample in a data set is taken after the 50th time point. The gene expression profiles are generated with a sigmoidal activator function.

### Preprocessing of B-cell lymphoma microarray data sets

The collection of the microarray gene expression data used in this study are from [21], which are accessible from the NCBI *Gene Expression Omnibus* (GEO) [34] (accession GSE2350). We denote the GSE2350 dataset that includes transformed and untransformed B-cell lymphoma samples as the *Basso GSE2350 dataset*. For our analysis we consider only samples for which raw gene expression data in form of CEL files are available. From the total of 387 samples of the GSE2350 dataset, 344 samples were available with raw CEL files. In the following, we call this data set *D*. The data set includes two Affymetrix chip platforms, *hgu95a* and *hgu95a_v2*. We used the mixture CDF environment hgu95av12mixcdf_1.0.tar.gz available from http://bmbolstad.com/misc/mixtureCDF/MixtureCDF.html to include only probe sets that have the same probe set annotation.

For a cross-dataset validation of our study, we preprocessed two additional B-cell lymphoma data set. We retrieved a Diffuse-large B-cell lymphoma *hgu133plus2* Affymetrix microarray data set with accession GSE11318 [27] including 203 samples, and a Diffuse large B-cell lymphoma *hgu133a* Affymetrix microarray data set with the accession GSE22470 [25] including 271 samples. These two data sets contain only untransformed B-cell lymphoma samples. We denote these as the *Lenz GSE11318 dataset* and the *Salaverria GSE22470 dataset*.

We processed all *CEL* files for each data set using RMA, a quantile normalization and summarization [35-37]. We extracted the *log*_2_ expression intensities for each probe set. Because a gene can be represented by more than one probe set, we calculate the median expression value for each gene by mapping the annotation of Affymetrix-ID to *Entrez* gene IDs to obtain a summary value for the genes. The *Basso GSE2350 dataset* comprises a total of 9,684 genes and 344 samples, where we do not exclude any unmapped probesets.

In order to perform a cross-dataset validation of the *Basso GSE2350 dataset*, we discarded all gene and probe set identifiers from the *Lenz GSE11318 dataset* and *Salaverria GSE22470 dataset* that are not present in the *Basso GSE2350 dataset*. After removal, the expression matrix of the *Lenz GSE11318 dataset* comprises 8,727 genes and 203 samples and the expression matrix of the *Salaverria GSE22470 dataset* comprises 8,664 genes and 271 samples.

### Gene regulatory network inference

We use the C3NET method [18] to infer the gene regulatory networks for the simulated gene expression data sets and the B-cell data. For each data set, a copula transformation is applied to the gene expression matrix as performed in [17]. Mutual information for all gene pairs is computed using the Pearson estimator [38,39]

(1) $$I(X,Y)=-\frac{1}{2}\log(1-\rho^{2}).$$

Here, *ρ* is the Pearson correlation coefficient. F-scores are estimated by

(2) $$F=\frac{2\mathrm{pr}}{p+r}.$$

Here, *p* is the precision and *r* the recall.

### Functional Analysis

The procedure for the Gene Ontology (GO) [40] enrichment analysis was implemented in R using the *Entrez* gene to GO annotation from the *hgu95a_v2* and the *org.Hs.eg.db* package and for the GO enrichment analysis the *topGO* package [41] from Bioconductor in R [42]. The significance level of the enrichment for a GO term was determined by a hyper-geometric test (Fisher’s Exact Test [43]). For the analysis, only terms assigned to more than 3 candidate genes are considered for the analysis.

### Network gene centrality pathway analysis

For the cross-dataset validation of the B-cell C3NET gene regulatory networks inferred from different data sets, we conducted a pathway-based network comparison. This method allows to identify functional subnetworks with the strongest structural similarities between pairs of gene regulatory networks.

We compare the underlying network structure between two C3NET gene regulatory networks, using the node betweenness centrality [44] measure. The node betweenness centrality for a gene *v*_*i*_ in a network is defined by [44]

(3) $$v_{i}=\underset{\mathrm{kl}}{\sum }\frac{n_{\mathrm{kl}}^{i}}{p_{\mathrm{kl}}}.$$

Betweenness centrality measures the proportion of all shortest paths between gene *v*_*k*_ and gene *v*_*l*_, which traverse gene *v*_*i*_ denoted by $n_{\mathrm{kl}}^{i}$, referred to all shortest paths between gene *v*_*k*_ and gene *v*_*l*_ denoted by *p*_*kl*_.

For two given gene regulatory networks, *G*_*a*_ and *G*_*b*_, we estimate the betweenness centrality values for all genes from a Gene Ontology (GO) term. Then, for each GO term, we perform Spearman’s rank correlation test [43] for the ranks of the betweenness centrality values. We adjust p-values using a FDR [45] correction for a given significance level of *α* = 0*.*05. For the analysis we use the Gene Ontology (GO) annotation from the Bioconductor *org.Hs.eg.db* package.

### Hierarchical network organization

In order to analyze the hierarchical organization of the B-cell C3NET gene regulatory network, we perform a three-step procedure based on bootstrap samples of the data. In the first step, we infer a network *G* by using all 344 samples of the microarray data set *D*. For this network, we identify its network components, which represent connected components. That means, from *G* we obtain a set of network components $풞=\{C_{1},\ldots ,C_{K}\}$ whereas *C*_*i*_ represents a list of genes that can be found in component *i*. These components have the property that for any pair of genes, e.g., *g*_*j*_, *g*_*k*_ ∈ *C*_*i*_ there exists a path connecting gene *g*_*j*_ with *g*_k_. However, for gene pairs from different components, e.g., *g*_*j*_ ∈ *C*_*i*_ and *g*_*k*_ ∈ *C*_*i′*_ there exists no path that connects these genes. The size of each network component is given by *N*_*i*_ = | *C*_*i*_ | , i.e., for example that network component *C*_*i*_ contains *N*_*i*_ genes. Here, *K* indicates the total number of components found in *G* we consider for our analysis. We would like to emphasize that the network components are naturally obtained because the inferred network *G* is usually an unconnected network, due to the conservative working principle of C3NET. Hence, $풞=\{C_{1},\ldots ,C_{K}\}$ correspond to these individual network components.In the second step, an ensemble of bootstrap data sets ${\left\{D_{i}^{B}\right\}}_{i=1}^{b}$ is generated from *D*, as described in Figure 2, from which we infer an ensemble of networks ${\left\{G_{i}^{B}\right\}}_{i=1}^{b}$, one for each bootstrap data set. Further, from the ensemble of networks, ${\left\{G_{i}^{B}\right\}}_{i=1}^{b}$, we estimate for each gene pair the fraction of inferred edges present in the ensemble,

(4) $$\begin{array}{lcrr} c_{\mathrm{ij}}=\frac{\left(\#e_{\mathrm{ij}}=1|\{G_{i}^{B}\}\right)}{b},\;\;(i,j)\in \{1,\ldots ,N\}. & & & \end{array}$$

This corresponds to the probability $\mathrm{Pr}(e_{\mathrm{ij}}=1|\{G_{i}^{B}\})$. Due to the fact that the underlying network *G* is undirected, *c*_*ij*_ is symmetric, i.e., *c*_*ij*_ = *c*_*ji*_.Finally, in the third step, the information obtained in step one and step two is combined by estimating the *average neighborhood closeness*, ${ĉ}_{kk^{\prime \prime }}$, from each network component *k* to network component *k’*, obtained the following way. A mean feature vector, ${ĉ}_{k}$, is estimated for each network component *k* by

(5) $$\begin{array}{lcrr} {ĉ}_{k}(i)=\frac{1}{N_{k}}\underset{j\in C_{k}}{\sum }c_{\mathrm{ij}},\;\;k\in \{1,\ldots ,K\}. & & & \end{array}$$

Here, *N*_*k*_ is the size of network component *k* and ${ĉ}_{k}(i)$ is the i-th component of the vector ${ĉ}_{k}$, which has length *N*. The interpretation of ${ĉ}_{k}(i)$ is *Pr(*component k is connected with gene i $\left|\{G_{i}^{B}\}\right)$. From this, the average neighborhood closeness between network component *k* and network component *k’* is obtained by

(6) $$\begin{array}{lcrr} {ĉ}_{kk^{\prime \prime }}=\frac{1}{N_{k^{\prime \prime }}}\underset{j\in C_{k^{\prime \prime }}}{\sum }c_{k}(j),\;\;k,k^{\prime \prime }\in \{1,\ldots ,K\}. & & & \end{array}$$

The interpretation of ${ĉ}_{kk^{\prime \prime }}$ is $\mathrm{Pr}(\text{component k is connected with component k’}|\{G_{i}^{B}\})$. Hence, the average neighborhood closeness ${ĉ}_{kk^{\prime \prime }}$ provides structural information about the involvement of individual genes between network component *k* and *k’* utilizing the variability within the data, as exploited by the bootstrap ensemble. We use the average neighborhood closeness ${ĉ}_{kk^{\prime \prime }}$ to define a *K* × *K* similarity matrix *U* by

(7) $$\begin{array}{lcrr} U_{kk^{\prime \prime }}={ĉ}_{kk^{\prime \prime }}. & & & \end{array}$$

For our analysis we are using *U* to define an error measure *d*_2_, defined in section ‘Graph edit distance hierarchy error’.

Due to the probabilistic interpretation of ${ĉ}_{kk^{\prime \prime }}$, which implies that $0\leq {ĉ}_{kk^{\prime \prime }}\leq 1$, these components are easily transformed into distance values by

(8) $$\begin{array}{lcrr} D_{kk^{\prime \prime }}=1-{ĉ}_{kk^{\prime \prime }}. & & & \end{array}$$

For our analysis we use the resulting *K* × *K* distance matrix *D* for a hierarchical clustering in combination with the “Ward” method. The overall procedure is summarized in Figure 5.

## Results

### Consistency of bootstrap ensembles

**Figure 3:**
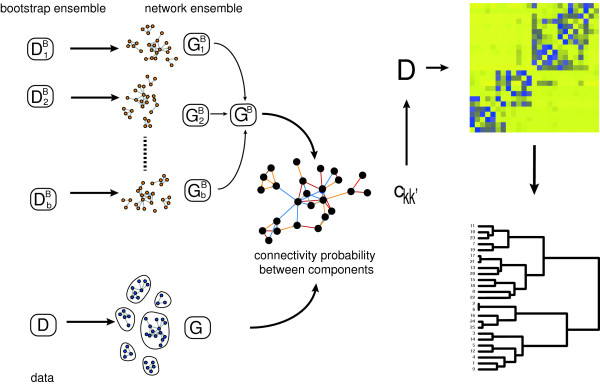
Illustration of the usage of the bootstrap ensemble for the clustering of the network components.

We start our analysis by performing simulations to compare the distributions of F-scores of an ensemble of independently generated data sets with two bootstrap ensembles. For an illustration of the generation of these bootstrap data and the difference between the three types of F-scores, see Figure 2. The colors of the arrows in this figure correspond to the colors of the boxplots shown in Figure 3. That means the blue boxplots correspond to F-scores obtained for an ensemble of 300 independently generated data sets. The red boxplots correspond to 30000 ( = 300 × 100) F-scores obtained by bootstrapping each of the 300 data sets 100 times. We call this bootstrap ensemble BE 1. The boxplots in green show the 300 averaged F-scores, i.e., each F-score is averaged over 100 bootstrap samples. We call this bootstrap ensemble BE 2. Figure 3 shows the distribution of these F-scores for scale-free networks in dependence of four different sample sizes.In general, one can see that the distributions of F-scores of the two bootstrap ensembles are similar in range, median and the interquartiles to the F-scores obtained for the ensemble of independently generated data sets. However, the F-scores for BE 1 (shown in red) contain some outliers. This can be expected, because the bootstrapping of the data leads in general to a loss of information, due to the fact that not all samples are available for the inference task. For this reason, the median F-scores decline slightly, as can be seen from Figure 3. However, this decline is rather moderate, e.g., compared to the overall increase for larger sample sizes. Further, there are only few outliers, indicating that only very few bootstrap data sets lead to atypical results. Hence, our analysis demonstrates that the usage of bootstrap ensembles leads to a good approximation compared to results for and ensemble of independently generated data sets. Due to the fact that the latter data are only available in simulation studies, but not for real biological data, a bootstrap ensemble is a valid approach to estimate the variability of the population of inferred networks from an ensemble of data sets. We repeated the above analysis for different network topologies, including random networks and directed acyclic graphs (not shown), and found qualitatively similar results as for the scale-free networks shown above.

These results demonstrate that the bootstrap data lead to very similar results as the independent data, independent of the sample size. Hence, in the specific context of network inference bootstrapping data is an efficient means to generate an ensemble of data to resemble an independently generated ensemble.

### Inferrability of a hierarchical organization

**Figure 6:**
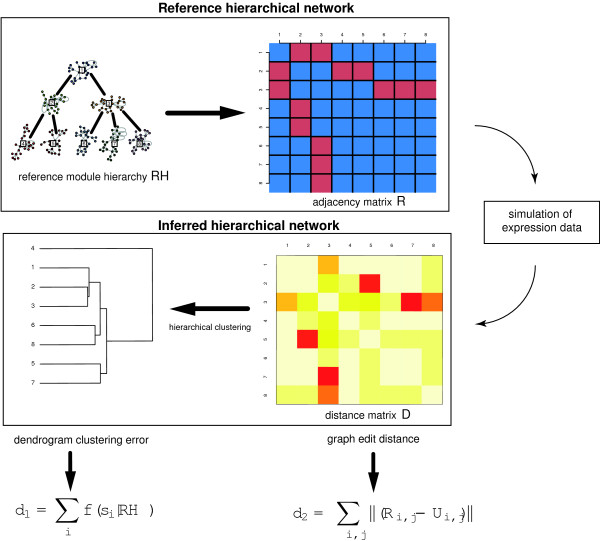
**Simulated gene expression data are generated from a network with a defined hierarchical structure** (***RH*).** From the simulated data, the distance matrix *D* is estimated. The inferred hierarchy of the network modules is obtained from a hierarchical clustering of matrix *D*. The measures *d*_1_ and *d*_2_ evaluate the estimated hierarchical structure.

Next, we evaluate the performance of our bootstrap approach for the inference of a network with a hierarchical structure. For this reason, we simulate gene expression data by using a network with a defined hierarchical organization of 8 interconnected MTM network modules, see Figure 4. The individual network modules are interconnected by 0, 3, 5, 10 and 15 edges. In total, we analyze gene expression data sets for 5 ensembles, each consisting of 100 data sets. In order to measure the performance of our bootstrap approach for the inference of the hierarchical organization of the network components from the simulated data we developed two measures. The first measure, *d*_1_, is the *dendrogram clustering error* that scores clustering errors of dendrogram splits between the true hierarchical structure and the inferred hierarchical structure (Figure 6). The second measure, *d*_2_, is the *graph edit distance hierarchy error* that computes the graph edit distance [46-48] between the reference adjacency matrix of interconnected network modules and the inferred similarity matrix *U*, described in section ‘Hierarchical network organization’.

**Figure 7:**
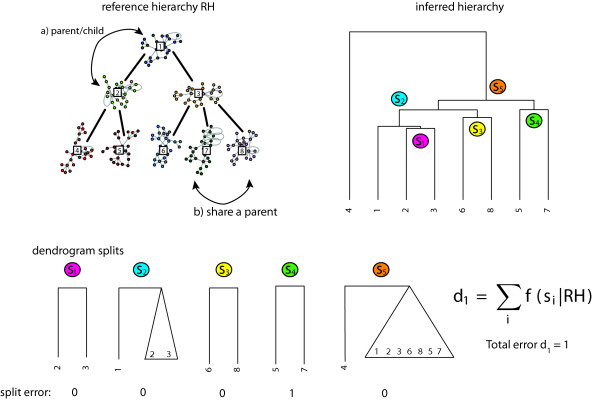
**Example for the functioning of***d*_1_. The reference hierarchy *RH* describes network modules that are directly connected by a parent/child relation or by network modules that share the same parent network module. The scoring function evaluates each cluster from the lowest to the highest split *s*_*i*_ in the dendrogram. In the given example, the inferred dendrogram contains a total of one clustering error (split *s*_4_).

We estimate for each simulated gene expression data set a distance matrix *D* and a dendrogram of the inferred network module hierarchy, obtained by application of the “Ward” method. A summary of this procedure is given in Figure 7.

#### Dendrogram clustering error

The dendrogram clustering error, *d*_1_, measures the number of clustering errors from the lowest to the highest split, *s*_i_, between the inferred hierarchical structure and the underlying true hierarchical reference structure *RH* (Figure 4). A split, *s*_i_, in the dendrogram describes either (a) a cluster of two network modules or (b) a cluster of network modules to a cluster of network modules. We calculate the clustering error *d*_1_ by

(9) $$d_{1}=\underset{i}{\sum }f(s_{i}|\mathrm{RH}).$$

**Figure 8:**
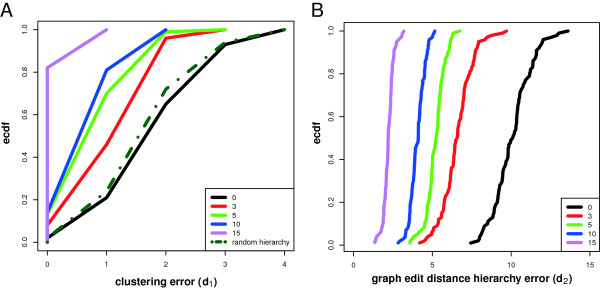
**Simulation study for the reconstruction of the hierarchical organization within a network.** Shown are the results for 100 simulated gene expression data sets generated from networks with network modules connected by 0, 3, 5, 10 and 15 edges. **A**) Dendrogram clustering error *d*_1_. **B**) Graph-edit distance hierarchy error.

The binary error function *f* (*s*_*i*_ | *RH*) ∈ {0, 1} scores the clustering error of a split as follows. Suppose, the reference hierarchy *RH* is resembled in split *s*_*i*_ for the cases (a) two clustered modules share the same parent module or (b) have a parent/child relationship. A clustering error of a split is counted as 1 if neither of the two cases is true, otherwise it is zero. For the case of a network module being clustered to a cluster of network modules, the relation (a) or (b) must be given to at least one network module of the cluster. The dendrogram distance *d*_1_ scores the total clustering error from the lowest to the highest split *s*_*i*_ in the dendrogram. The maximal number of clustering errors is the total number of splits defined in the dendrogram. An example for the calculation of the error score *d*_1_ is shown in Figure 6. In order to obtain a null model of the dendrogram clustering error that corresponds to the random clustering of network modules, we generate reference networks with permuted module labels. That means, we assume a network with a modular structure as shown in Figure 4, but permute the labels of the corresponding modules within this network.In Figure 8 A we show the empirical cumulative distribution function (ecdf) of the dendrogram clustering error *d*_1_ for a variety of networks with different numbers of module interconnecting edges. For the null model, that means for networks resembling a random hierarchy structure as defined above, only about 20% of the cases reach a clustering error with ≤ 1. This is similar to the results obtained for networks with no interconnections between modules (black). Interestingly, already for 3 connections between modules (red) we observe that 40% of these networks have a clustering error ≤ 1. The results for 5 (green) and 10 (blue) module interconnecting edges show 70% and 80% with a clustering error ≤ 1. Finally, for 15 module interconnecting edges all hierarchy networks can be recovered with *d*_1_ ≤ 1.

#### Graph edit distance hierarchy error

The graph edit distance hierarchy error, *d*_2_, is a measure for the error of the inferred module hierarchy. The reference hierarchy is described by the adjacency matrix *R* for the modules. Here an entry of *R*_*ij*_ = 1 denotes that the two network modules *i* and *j* are connected. We measure the distance between the true underlying hierarchy *R* and the inferred similarity matrix *U*, given by Eq. 7, by

(10) $$d_{2}=\underset{i,j}{\sum }∥(R_{i,j}-U_{i,j})∥.$$

In Figure 8 B we show the the empirical cumulative distribution function (ecdf) of the graph edit distance hierarchy error *d*_2_. The values of *d*_2_ decrease with an increasing number of interconnecting edges between the network modules. This means adding edges between the network modules helps in reducing the inference error. For the networks with no interconnections between the network modules (black) *d*_2_ is largest, as expected. These results correspond to the absence of a hierarchy between the network modules. Overall, the results for *d*_2_ are similar to *d*_1_ demonstrating that regardless of the chosen error measure a relatively low number of interconnecting edges is sufficient to enable the recovery of at least parts of the present hierarchy in the network.

### Analyzing network components of the B-cell C3NET gene regulatory network

**Figure 9:**
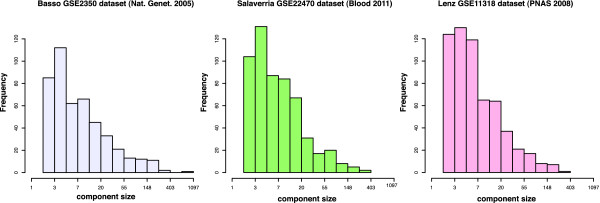
**Histogram of the size of the 463 separate network components of the inferred B-cell C3NET gene regulatory network inferred from the*Basso GSE2350 dataset*(left).** Middle and right: DLBCL-C3NET gene regulatory networks with 556 separated network components for the *Salaverria GSE22470 dataset* and 593 separated network components for the *Lenz GSE11318 dataset*.

We infer a C3NET [18] gene regulatory network from 344 samples of the B-cell lymphoma microarray gene expression data set from [21]. The resulting B-cell C3NET gene regulatory network comprises 9,684 genes and 9,221 edges distributed over 463 separate network components (with > 1 gene). For a cross-dataset validation, we further inferred two additional C3NET gene regulatory networks from the DLBCL data sets in [25,27]. The DLBCL-C3NET gene regulatory network of the *Lenz GSE11318 dataset* comprises 8,727 genes and 8,134 edges and the DLBCL-C3NET gene regulatory network of the *Salaverria GSE22470 dataset* comprises 8,664 genes and 8,108 edges. Figure 9 shows a summary of the size distributions of the inferred network components.

For the B-cell C3NET gene regulatory network, the *K* = 25 largest network components (5% right quantile) have > 100 genes and comprise a total of 4,673 genes representing 48% of all genes in the network. The giant connected component consists of 884 genes and 883 edges. For the two DLBCL-C3NET gene regulatory networks the largest *K* = 25 network components of the inferred networks comprise 3,331/3,477 genes representing 38%/40% of all genes in the entire gene regulatory network. The giant connected components of the two DLBCL gene regulatory networks consist of 299/395 genes and 298/394 edges.

### Functional Network Analysis

In order to obtain a biological interpretation of the inferred B-cell C3NET gene regulatory network, we perform a Gene Ontology [40] enrichment analysis for each of the *K* = 25 largest network components. To perform this analysis, the inferred network component are used to define gene lists for which we perform an enrichment analysis.The Tables 1,2 and 3 present results for the giant connected component. In these tables, the top 15 enriched GO terms with a significant p-value ≤ 5*e*^− 4^ are shown. The three tables correspond to the Gene Ontology categories *Biological Process* (Table 1), *Molecular Function* (Table 2) and *Cellular Component* (Table 3). The genes in the giant connected component show an enrichment in biological processes for G-protein-coupled-receptor protein signaling pathway (89 genes), cell-cell signaling (87 genes) and calcium ion transport (26 genes) (Table 1). The cellular component analysis shows an enrichment, e.g., for plasma membrane proteins (264 genes), ion channel complexes (125 genes) and cell junction proteins (48 genes) (Table 3). The molecular function analysis shows an enrichment, e.g., for G-protein coupled receptor activity (60 genes) and ion channel activity (38 genes) (Table 2).

**Table 1:** GO category Biological Process: Enrichment analysis of the genes in the giant connected component

GO ID	Term	All	Sig	Exp	p-value
GO:0007186	G-protein coupled receptor protein signaling pathway	450	89	38.65	1.4e-14
GO:0050877	neurological system process	567	99	48.70	1.4e-12
GO:0032501	multicellular organismal process	2714	317	233.12	1.7e-12
GO:0003008	system process	858	127	73.70	1.2e-10
GO:0007267	cell-cell signaling	532	87	45.70	1.3e-09
GO:0007166	cell surface receptor linked signaling pathway	1071	145	91.99	2.9e-09
GO:0019226	transmission of nerve impulse	312	58	26.80	9e-09
GO:0023033	signaling pathway	1127	148	96.80	1.6e-08
GO:0023052	signaling	2504	279	215.08	3.4e-08
GO:0023060	signal transmission	2154	246	185.02	4.6e-08
GO:0023046	signaling process	2155	246	185.10	4.9e-08
GO:0030001	metal ion transport	281	51	24.14	1.6e-07
GO:0007268	synaptic transmission	274	50	23.54	1.8e-07
GO:0070838	divalent metal ion transport	114	27	9.79	8.1e-07
GO:0006816	calcium ion transport	112	26	9.62	2e-06

**Table 2:** GO category Molecular function: Enrichment analysis of the genes in the giant connected component

GO ID	Term	All	Sig	Exp	p-value
GO:0004930	G-protein coupled receptor activity	247	60	21.35	5.2e-14
GO:0004872	receptor activity	797	121	68.89	1.1e-10
GO:0004888	transmembrane receptor activity	547	88	47.28	3.3e-09
GO:0004871	signal transducer activity	1118	150	96.63	4.6e-09
GO:0060089	molecular transducer activity	1118	150	96.63	4.6e-09
GO:0005509	calcium ion binding	528	85	45.64	6.2e-09
GO:0046873	metal ion transmembrane transporter activity	166	35	14.35	4.8e-07
GO:0042165	neurotransmitter binding	51	17	4.41	6.2e-07
GO:0005261	cation channel activity	138	30	11.93	1.6e-06
GO:0005216	ion channel activity	198	38	17.11	1.9e-06
GO:0022836	gated channel activity	176	35	15.21	2e-06
GO:0015267	channel activity	215	40	18.58	2.3e-06
GO:0022803	passive transmembrane transporter activity	215	40	18.58	2.3e-06
GO:0022857	transmembrane transporter activity	510	74	44.08	4.2e-06
GO:0022838	substrate-specific channel activity	206	38	17.81	5e-06

**Table 3:** GO category Cellular component: Enrichment analysis of the genes in the giant connected component

GO ID	Term	All	Sig	right	p-value
GO:0016021	integral to membrane	2201	282	193.28	2.2e-14
GO:0031224	intrinsic to membrane	2260	285	198.46	1.2e-13
GO:0044459	plasma membrane part	1388	195	121.89	4e-13
GO:0044425	membrane part	2739	327	240.53	1.1e-12
GO:0005886	plasma membrane	2084	264	183.01	1.3e-12
GO:0005887	integral to plasma membrane	900	131	79.03	9e-10
GO:0031226	intrinsic to plasma membrane	915	132	80.35	1.4e-09
GO:0016020	membrane	3462	372	304.02	4.3e-08
GO:0034702	ion channel complex	125	31	10.98	6.7e-08
GO:0005576	extracellular region	1077	136	94.58	3e-06
GO:0034703	cation channel complex	80	21	7.03	3.4e-06
GO:0034704	calcium channel complex	22	10	1.93	6.2e-06
GO:0005903	brush border	31	11	2.72	3.6e-05
GO:0005891	voltage-gated calcium channel complex	17	8	1.49	4e-05
GO:0030054	cell junction	327	48	28.72	0.00024

**Figure 10:**
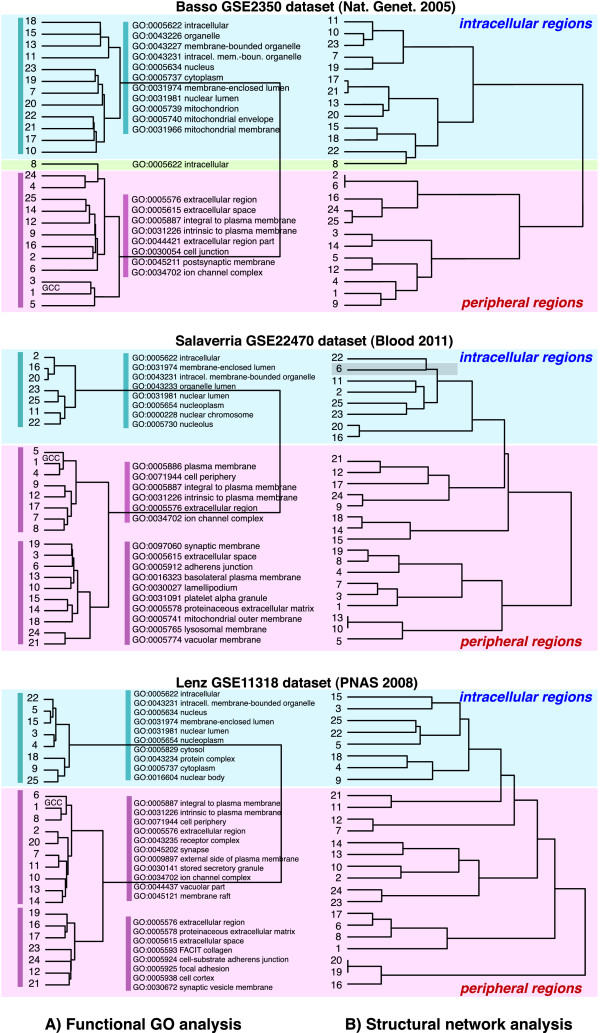
**Hierarchical organization of the*K*= 25 largest network components of the B-cell C3NET gene regulatory network inferred from the*Basso GSE2350 dataset*and the DLBCL gene regulatory network inferred from the***Salaverria GSE22470 dataset***and the*Lenz GSE11318 dataset*.** The numbers of the network components correspond to the leaves of the dendrogram. The GCC corresponds to number ‘1’. **A)** Clustering of the GO enrichment analysis, category *Cellular Component*. We show GO terms from the top ranked frequency counts of GO terms for the branches, indicted by the vertical color bars. **B)** Hierarchical clustering of the structural network components. Left and right: The red part of a dendrogram represents network components with Gene Ontology terms enriched with extracellular and membrane proteins. Green and blue network components are enriched with intracellular proteins.

To study the biological relation and functional diversity between the individual network components, we cluster the network components according to the results of the Gene Ontology enrichment analysis from the category *Cellular Component*. Specifically, we conduct a clustering analysis of GO terms for the *K* = 25 network components in the following way. From testing 1,020 GO terms of the category *Cellular Component* we find that 529 of these test at least for one network component significant.The functional hierarchical clustering of the network components is generated from the overlap of significant Gene Ontology terms between all pairwise comparisons of the Gene Ontology enrichment analysis of the individual network components. A pairwise distance matrix is computed from the shared number of Gene Ontology terms for a significance level of *α* = 0*.*05. For the hierarchical clustering we use the “Ward” method, see Figure 10 A (first column).

The numbers of the leaves in the dendrogram correspond to the rank-labels of the network components, whereas ‘1’ corresponds to the GCC. The provided GO terms correspond to the most frequently enriched terms found in the corresponding branches of the dendrogram. The hierarchical clustering based on the functional GO analysis of the network components separates the dendrogram into two principle branches. The first branch, shown in red, consists of highly enriched extracellular proteins, intrinsic and integral membrane proteins, cell junction and ion channel complex proteins. The second branch, shown in blue, is highly enriched for intracellular proteins from the nucleus, mitochondrion and cytoplasm.

In order to provide a comparison with the gene regulatory networks inferred from the two DLBCL gene expression data sets, we perform the same analysis for the *Lenz GSE11318 dataset* and the *Salaverria GSE22470 dataset* (Figure 10). The network components of the DLBCL gene regulatory networks show a similar bipartition as observed for the *Basso GSE2350* gene regulatory network separating into two principle branches for the peripheral and the intracellular regions of the cell. A major difference is that the DLBCL gene regulatory networks show a bipartition within the principle branch enriched with genes of the peripheral regions.

### Hierarchical organization of the B-cell C3NET gene regulatory network

Next, we study the hierarchical organization of the *K* = 25 largest network components of the B-cell gene regulatory network. This analysis is similarly conducted as for the simulated data, described in section ’Inferrability of a hierarchical organization’. That means, first, we generate *b* = 100 bootstrap data sets from which we infer an ensemble of networks ${\left\{G_{i}^{B}\right\}}_{i=1}^{b=100}$. Then, we determine from these networks a distance matrix *D*, which we use for a hierarchical clustering. As agglomeration clustering method we use again the “Ward” method.The resulting dendrograms are shown in Figure 10 B (second column). Also in these dendrograms, the rank-labels of the network components correspond to the leaf labels. As for the clustering of significantly enriched GO terms between the individual network components, we observe a bifurcation into two principal branches. Though the subgroupings of individual components differ to some extend in the respective categories, one can see that the same two principal branches are obtained as for the clustering of the GO terms in Figure 10 A. The first branch corresponds to the extracellular and membrane intrinsic proteins enriched network components and the second branch belongs to intracellular network components enriched by genes in the nucleus, mitochondria and cytoplasm.

We would like to emphasize that the generation of both dendrograms is based on complementary information. Figure 10 A is obtained from dissimilarity values among GO terms, not considering the inferred interactions among genes. In contrast, Figure 10 B is obtained from a structural analysis of the inferred network, not considering GO terms. This demonstrates that the extracted information from two complementary analysis methods leads to coinciding information with respect to the principle separation of cellular components of a biological cell.

Further, we compare the results of the B-cell C3NET gene regulatory network to the DLBCL-C3NET gene regulatory networks inferred from the *Lenz GSE11318 dataset* and the *Salaverria GSE22470 dataset* (Figure 10 B). Although the subgroupings between the functional and structural hierarchical clustering differ to some extend, overall, the network components of the two DLBCL gene regulatory networks show a similar clustering into two major branches of peripheral and intracellular regions. However, the bipartiton of the structural network components (second column in Figure 10) is less pronounced as observed for the B-cell C3NET gene regulatory network for the *Basso GSE2350 dataset*.

### Identification of novel key signaling pathways in B-cell lymphoma

Hub genes of the B-cell C3NET gene regulatory network are genes with the largest node degree among all genes in the network. Intuitively, such genes are the most interesting targets to study as they are more likely to be associated with multiple pathways, e.g., signaling pathways and thus form putative key regulators for a large diversity of biological processes.

From the entire B-cell C3NET gene regulatory network, we extracted the largest 25 hub genes with more than 20 connections. In Table 4 we give an overview of these hub genes including their gene identifiers and a selected GO term in order to facilitate the interpretation of their functional context. The selected hub genes play crucial roles in signaling processes such as receptors, ion channels and transporters, cell adhesion proteins and transcription factors. To our knowledge these genes were not studied in B-cell lymphoma to date (Table 4).

**Table 4:** **Top 25 hub genes with a degree (deg) larger than 20 found in the B-cell lymphoma gene regulatory network - genes are described by their** ***Entrez*** **gene id, gene symbol, and, if available, one selected annotation term from GO (category Biological Process), bc refers to the betweenness centrality and the number in brackets to its rank with respect to the bc values**

Entrez	Symbol	deg/bc(rank)	Description
778	CACNA1F	46/282019(1)	calcium channel, voltage-dependent, L type, alpha 1F subunit (calcium ion transport GO:0006816)
2949	GSTM5	38/14454(39)	glutathione S-transferase mu 5 (metabolic process GO:0008152)
7275	TUB	37/16818(35)	tubby homolog (mouse) (response to stimulus GO:0050896)
9080	CLDN9	35/65406(6)	claudin 9 (calcium-independent cell-cell adhesion GO:0016338)
3363	HTR7	33/1436(406)	5-hydroxytryptamine (serotonin) receptor 7 (adenylate cyclase-coupled) (signal transduction GO:0007165)
1579	CYP4A11	32/24724(25)	cytochrome P450, family 4, subfamily A, polypeptide 11 (long-chain fatty acid metabolic process GO:0001676)
796	CALCA	32/46142(11)	calcitonin-related polypeptide alpha (endothelial cell proliferation GO:0001935)
7546	ZIC2	28/6978(95)	Zic family member 2 (odd-paired homolog, Drosophila) (cell differentiation GO:0030154)
1993	ELAVL2	28/9991(62)	ELAV (embryonic lethal, abnormal vision, Drosophila)-like 2 (Hu antigen B) ( NA)
1549	CYP2A7	27/33469(17)	cytochrome P450, family 2, subfamily A, polypeptide 7 (oxidation-reduction process GO:0055114)
5554	PRH1	27/8093(77)	proline-rich protein HaeIII subfamily 1 ( NA)
2516	NR5A1	27/248679(2)	nuclear receptor subfamily 5, group A, member 1 (cell-cell signaling GO:0007267)
11222	MRPL3	27/12748(45)	mitochondrial ribosomal protein L3 (translation GO:0006412)
92017	SNX29	26/159597(3)	sorting nexin 29 (cell communication GO:0007154)
6534	SLC6A7	25/46858(10)	solute carrier family 6 (neurotransmitter transporter, L-proline), member 7 (proline transport GO:0015824)
115703	ARHGAP33	25/28006(21)	Rho GTPase activating protein 33 (signal transduction GO:0007165)
40	ACCN1	24/5548(113)	amiloride-sensitive cation channel 1, neuronal (sodium ion transport GO:0006814)
1943	EFNA2	23/34712(16)	ephrin-A2 (cell-cell signaling GO:0007267)
7047	TGM4	23/147229(4)	transglutaminase 4 (prostate) (peptide cross-linking GO:0018149)
343	AQP8	22/17440(34)	aquaporin 8 (water transport GO:0006833)
9127	P2RX6	22/63918(7)	purinergic receptor P2X, ligand-gated ion channel, 6 (signal transduction GO:0007165)
2797	GNRH2	21/2976(202)	gonadotropin-releasing hormone 2 (signal transduction GO:0007165)
5545	PRB4	21/9246(66)	proline-rich protein BstNI subfamily 4 ( NA)
4706	NDUFAB1	21/11907(49)	NADH dehydrogenase (ubiquinone) 1, alpha/beta subcomplex, 1, 8kDa (electron transport chain GO:0022900)
553	AVPR1B	21/5080(130)	arginine vasopressin receptor 1B (signal transduction GO:0007165)

**Figure 11:**
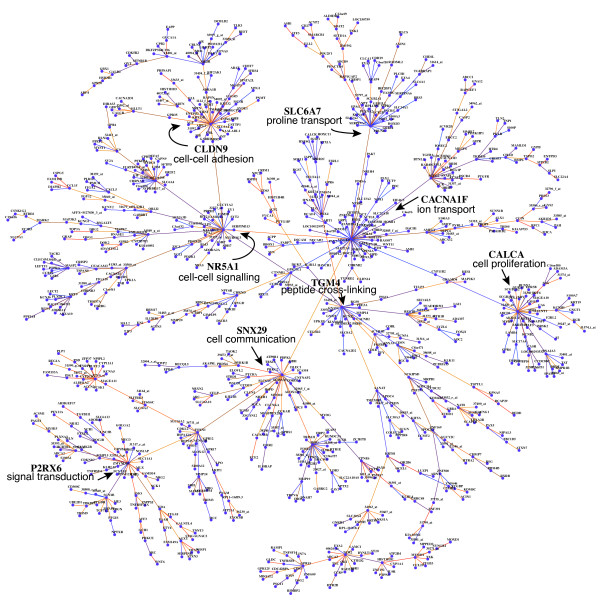
**The giant connected component consists of 884 genes.** The ensemble support values of the individual edges are shown from red (high support) to blue (low support). The 7 largest hub genes of the giant connected component are highlighted, and a GO term from the category Biological Process is included: *CACNA1F* (46),*CLDN9* (35), *CALCA* (32), *NR5A1* (27), *SNX29* (26), *SLC6A7* (25) and *TGM4* (23) and *P2RX6* (22). Here, the number in bracket corresponds to the number of direct gene neighbors.

The structure of the *giant connected component* (GCC) network consists of small, interconnected network modules with intramodular hub genes consisting in total of 884 genes and 883 edges (Figure 11). The GCC contains the largest hub gene (*CACNA1F*) of the entire B-cell C3NET gene regulatory network, and in total 7 of the top-ranked 25 hub genes (Figure 11). We highlighted the hub genes in the GCC network in Figure 11 and find that these are involved in adhesion, signaling and proliferation processes. In the following, we discuss some of these hub genes in more detail. The largest hub gene, with a total of 46 connections, is the calcium channel subunit *CACNA1F*. This gene belongs to the class of voltage gated calcium channels that regulate calcium influx and intracellular processes such as signaling pathways and gene expression. In particular, *CACNA1F* was found to be highly expressed in human lymphoid tissues such as plasma cells within germinal centers and therefore assumed to play a role in immune responses [49]. *CLDN9* (35 gene neighbors) is a member of tight junction proteins which establish a permeability barrier between cells that play also a role in transport and signalling processes and have also been observed to be important for HPC virus entry[50]. *CALCA* (32 gene neighbors) calcitonin lowers calcium concentrations and plays a role in adhesion, cell migration and cell differentiation [51]. *NR5A1* (27 gene neighbors) is a transcription factor that regulates cell growth, cell differentiation and developmental processes [52]. *SNX29* (26 gene neighbors) belongs to a protein family involved in protein membrane trafficking that have a variety of protein motif binding domains [53].

### Influence of activator and repressor links

In this section we study the inferrability of activator and repressor links. First we determine the correlation coefficient of all significant edges in the inferred network and obtain their corresponding p-values from testing for a vanishing Pearson correlation coefficient. Second, we conduct a multiple testing correction using the Benjamini-Hochberg procedure [45]. The edges that are statistically significant are identified as activator correspondingly repressor edges if the sign of the correlation coefficient is positive respectively negative.

In the inferred B-cell C3NET gene regulatory network, we identify a total of 847 repressor edges and 8,372 activator edges. The estimated true reconstruction rate for repressor and activator edges is obtained from the bootstrap ensemble. A two-sample Kolmogorov-Smirnov test [43] comparing the distributions of the true reconstruction rates indicates a significant difference between these two distributions with a p-value of *p* = 2*.*2 × 10^− 16^. Further, we find that activator edges are easier to infer than repressor edges, because activator edges have statistically a higher true positive rate than repressor edges.

### Relationship of node degrees in the gene regulatory network and gene expression values

Next, we investigate the node degrees of genes in the inferred B-cell C3NET gene regulatory network and compare these with the variances of their gene expression values. We perform a *loess* (locally weighted scatterplot smoothing) [54] regression on the logarithm of the variances of the gene expression values and the corresponding node degree for each gene. We observe a positive correlation for genes up to a node degree of 7. In contrast, genes with a higher node degree show a negative correlation (results not shown). Thus, genes with a higher node degree in the inferred B-cell C3NET gene regulatory network show a smaller variation in their expression profile among the different samples of the expression data set.

Similarly, the connection between the gene expression variation and the node degrees in a protein-protein network was studied in [55]. There it was shown that with an increasing degree of the proteins, the gene expression variation decreases. Hence, for degrees larger than 7, both results coincide, however, for smaller degrees there seem to be differences between a protein-protein network and a gene regulatory network.

### Cross-dataset validation for cellular component subnetworks

We perform a cross-dataset validation studying the structural similarity of our B-cell C3NET gene regulatory network with two additional DLBCL-C3NET gene regulatory networks we inferred from observational germinal center tumor data sets from [27] (*Lenz GSE11318 dataset*) and [25] (*Salaverria GSE22470 dataset*). In order to assess the structural similarity between networks, we use the (vertex) *betweenness centrality* measure [44] in combination with Spearman’s rank correlation coefficient [43]. We use Spearman’s rank correlation coefficient to test if structural components of two networks are similar to each other with respect to the order of the *vertex betweenness centrality* values of the genes. Specifically, in the following, we study two different scales of the networks. First, we compare the entire networks using all genes. This corresponds to a *global* comparison. Second, we compare subnetworks defined as *cellular components* according to the gene ontology database. This corresponds to a *local* comparison.

From the global comparison, we find that the B-cell C3NET gene regulatory network shows a significant correlation of *r* ∼ 0*.*12 ( *p* ≤ 2*.*2^− 16^) to the DLBCL-C3NET gene regulatory networks of the *Salaverria GSE22470 dataset* and *r* ∼ 0*.*14 ( *p* ≤ 2*.*2^− 16^) to the DLBCL-C3NET gene regulatory network of the *Lenz GSE11318 dataset*. A comparison between the two DLBCL-C3NET gene regulatory networks shows also a significant correlation of *r* ∼ 0*.*24 ( *p* ≤ 2*.*2^− 16^).For the local comparisons, we test a total of 435 *cellular components* (corresponding to gene sets) that can be found in the networks having more than 10 genes. From these cellular components, we identify the ones with a statistically significant Spearman rank correlation coefficient between profile vectors whose components correspond to the *vertex betweenness centrality* values of the genes in cellular components. To the resulting nominal p-values, we are applying the Benjamini-Hochberg multiple testing correction procedure [45] to control the FDR at a level of 5%.

From the comparisons of the B-cell C3NET gene regulatory network with the DLBCL-C3NET gene regulatory network obtained from the *Lenz GSE11318 dataset*, we identify 95 (21%) gene sets, and for the comparison of the B-cell C3NET gene regulatory network with the DLBCL-C3NET gene regulatory networks obtained from the *Salaverria GSE22470 dataset*, we find 72 (16.5%) gene sets with a statistically significant correlation. In total, 58 terms are simultaneously significant in both network comparisons. These terms involve the *basal part of cell*, *cell periphery*, *endosome* and 17 gene sets sharing the parental term *GO:0032991 macromolecular complex*, e.g., *histone mehyltransferase complex*, *anaphase-promoting complex*, *ribosome* and *cation chanel complex*. In Table 5, we show the 30 Gene Ontology *cellular component* gene sets with the highest structural similarity between the B-cell C3NET gene regulatory network and the two DLBCL-C3NET gene regulatory networks. Each of the presented terms is statistically significant in both comparisons and the subscript ‘*ave*’ indicates the averaged values over these two comparisons.

**Table 5:** **Network similarity analysis for cellular components between the B-cell C3NET gene regulatory network and the Lenz and Salaverria gene regulatory network for 30 from the 58 cellular component subnetworks with the highest correlation coefficient of the betweenness centrality, significant in both comparisons - the columns denote the size (number of genes) of a Gene Ontology term represented in the subnetworks, betw**_***avg***_**the average betweenness for the two comparisons, *r***_***avg***_**Spearman’s rank correlation coefficient and** ***p***_***avg***_**the FDR adjusted p-value**

GOID	Term	Size	betw_*avg*_	*r*_*avg*_	pval_*avg*_(FDR)
GO:0045178	basal part of cell	47	230	0.51	1.83e-03
GO:0035097	histone methyltransferase complex	126	187	0.37	1.98e-02
GO:0031967	organelle envelope	109	212	0.34	4.20e-03
GO:0034361	very-low-density lipoprotein particle	67	183	0.33	2.45e-02
GO:0055037	recycling endosome	82	167	0.31	1.38e-02
GO:0034364	high-density lipoprotein particle	169	172	0.28	2.23e-04
GO:0031461	cullin-RING ubiquitin ligase complex	429	183	0.25	1.17e-04
GO:0030017	sarcomere	325	235	0.24	7.26e-03
GO:0044422	organelle part	350	167	0.21	4.00e-04
GO:0005680	anaphase-promoting complex	295	156	0.20	6.33e-04
GO:0044441	cilium part	654	355	0.20	4.17e-07
GO:0005788	endoplasmic reticulum lumen	368	245	0.20	6.16e-03
GO:0044445	cytosolic part	625	354	0.19	3.01e-06
GO:0005665	DNA-directed RNA polymerase II, core complex	640	264	0.19	5.20e-05
GO:0005789	endoplasmic reticulum membrane	656	247	0.18	8.14e-06
GO:0005903	brush border	2234	414	0.17	2.09e-06
GO:0044432	endoplasmic reticulum part	983	410	0.17	1.43e-07
GO:0005765	lysosomal membrane	1894	338	0.17	2.12e-04
GO:0034703	cation channel complex	923	277	0.17	7.43e-07
GO:0071944	cell periphery	290	198	0.16	1.10e-02
GO:0035085	cilium axoneme	1586	261	0.16	2.62e-10
GO:0005769	early endosome	1585	329	0.16	1.76e-09
GO:0005773	vacuole	1585	285	0.16	2.62e-10
GO:0005768	endosome	1585	324	0.16	1.56e-02
GO:0044309	neuron spine	708	317	0.16	8.22e-05
GO:0044440	endosomal part	3021	287	0.16	1.44e-05
GO:0000313	organellar ribosome	1545	240	0.16	6.90e-04
GO:0005669	transcription factor TFIID complex	1480	308	0.15	5.94e-09
GO:0008305	integrin complex	1609	307	0.15	3.14e-09
GO:0008180	signalosome	1272	245	0.15	1.56e-02

## Discussion

In this article, we inferred a B-cell gene regulatory network from B-cell lymphoma gene expression data [21] using the C3NET algorithm [18]. We found that the inferred B-cell C3NET gene regulatory network is characterized by individual network components that are organized by smaller interconnected network modules with intramodular hub genes. Further, we found that the giant connected component of the network is composed of 884 genes which show a significant enrichment for plasma membrane proteins that are involved in G protein signaling pathways and ion channel complexes. From the literature, it is known that ion channels play a key role for the signal transduction mechanism in lymphocytes [56]. Additionally, we found that the 25 largest components of the entire network can be categorized into two major classes. The first class, including the largest network component, is enriched by genes that are located at the membrane and the extracellular space at the physical periphery of the cell whereas the second class comprises network components located in the intracellular organelles such as in the cytoplasm, nucleus and mitochondrion. Further, the hub genes of the B-cell C3NET gene regulatory network were identified to play crucial roles in cell signaling, adhesion and cell proliferation processes.

It is believed that B-cell lymphoma subtypes show characteristic gene expression profiles of B-cells that are arrested in specific developmental stages [57]. The emergence of a lymphoma phenotype is thus understood to result from an impairment of pathways that control B-cell differentiation, proliferation and apoptosis processes [57]. The organizational structure of gene regulatory networks is a rich source of information to study specific molecular mechanisms of B-cell lymphoma. However, the combination of observational and experimental conditions from a variety of different B-cell lymphoma, including transformed and untransformed cells, as for our data [21], does not allow to infer a gene regulatory network for one particular subtype of B-cell lymphoma. Thus we are of the opinion that our inferred B-cell C3NET gene regulatory network represents an *average* representation of B-cell lymphoma reflecting different phenotypic subtypes with which the information conveyed by the gene expression values is associated.

In [18] it has been demonstrated that *not* all regions within a network can be inferred with the same inference accuracy. That means, the inference of networks is heterogeneous with respect to distinct edges in the network. It has been shown that moderately interconnected genes are easier to infer. This corresponds to the edges of linearly connected genes and the edges toward the leaf nodes of the network that are at the ’periphery’ of the network. The results in [18] have been obtained for simulated data. However, for a real biological gene regulatory network it was unclear what genes correspond to the periphery of this cellular network. In contrast, in this paper we demonstrated that the periphery of the inferred B-cell C3NET gene regulatory network is centered around transmembrane proteins and the linear parts of the gene regulatory network correspond to signaling pathways and transmembrane receptor or ion channel proteins involved in signaling cascades. We would like to note that these transmembrane proteins could form putative drug targets for B-cell lymphoma.

The C3NET algorithm selects at most one edge for each gene, having maximum mutual information value. Therefore, this algorithm intends to capture the *conservative causal core* of the whole regulatory network only. This is in contrast to many other network inference methods [17,21,58]. For this reason, it is no surprise that a previous analysis of the same data set employing a different network inference method [17] found that their inferred regulatory network is governed by major hub genes, which mark key regulators such as transcription factors [21]. In particular, the network inferred by ARACNE consisted of 129,000 edges and their major hub genes are reported to be cell cycle regulators. In contrast to these results, we found by our analysis a network with 9, 684 genes and 9, 221 edges enriched for signaling pathways and transmembrane receptors characterizing the physical periphery of a cell rather than its nucleus. From this and the conservative characteristics of C3NET, we conclude that the strongest signal within the data set [21] is actually from signaling pathways rather than from transcription regulation. Doubtlessly, the later is present too, however, with a reduced strength.

Another difference to the study in [21] is that we introduced in this article a novel bootstrap approach to reveal the *hierarchical organization* of the B-cell C3NET gene regulatory network. Due to the inferential characteristics of C3NET the resulting network inferred from using all 344 microarray samples resulted in several separate network components which we used to define network modules. That means, there is no need to apply module finding algorithms [59-61] but we obtain such modules naturally by the application of C3NET. In order to infer the hierarchical organization of these modules, we utilized a bootstrap ensemble, from which we estimated an ensemble of networks. Combining the ensemble of these networks with the information about the network components obtained from the complete data set, allowed us to obtain a structural clustering reflecting the hierarchical organization of these network components. We would like to emphasize that this hierarchical clustering does not utilize information about GO terms. This is in contrast to the hierarchical clustering of GO terms presented in Figure 10 A. Nevertheless, we identified the major branches in Figure 10 B that correspond well to the clustering of the GO terms in Figure 10 A. We would like to indicate that our results confirm findings presented in [15]. It was found that the yeast and the *E. coli* protein network can be separated into two highly modular subnetworks which showed a functional enrichment for intracellular and extracellular processes. Hence, this may hint to a fundamental organization scheme of cellular networks. A potential hypothesis derived from these results is that the hierarchy among the network components may reflect aspects of the information flow between these components [62,63].

There are several advantages resulting form our approach, we would like to highlight. First, our investigation of the hierarchical organization of the B-cell C3NET gene regulatory network is at the abstraction level of *network components* or modules, but not *genes*. As such it resembles a systems approach [64-66]. This leads to a tremendous reduction in the complexity of the problem, and specifically in the interpretation of the obtained dendrograms shown in Figure 10. Second, on a technical note the size of the bootstrap ensemble was chosen large enough so that a further increase in its size does not lead to a modification of the obtained clustering. For this reason, the obtained results are stable. Third, the merit of bootstrapping is well known in many branches of statistics [22,67], where it is frequently used to quantify the variability within the data. In our approach, we utilize the data variability by exploiting mutual information values which are too weak in the whole data set to either pass a statistical test or which are not the maximum mutual information value for any gene. For example, there may be genes that have several significant interactions with other genes within a very small margin. For such cases, the bootstrapping allows to favor different gene pairs, because a slight change in the constitution of a data set may lead to alternating selections regarding the maximum mutual information valued gene pair.

Finally, we would like to note that results from our reanalysis of the data set [21] demonstrate that the biological information buried within large-scale high-throughput data is rich allowing to investigate a multitude of different biological questions.

## Conclusions

With the increasing quality of network inference algorithms, we are heading toward the next major challenge we are facing in the post-genomic era, namely: What do the inferred networks mean? An analysis of the hierarchical organization of a network is just one aspect thereof, but we think, an import one. Due to the fact that one can study the hierarchy among genes, pathways, subnetworks or combinations thereof the complexity of this problem might be unprecedented. The bootstrap approach presented in this paper represents a simple, yet, flexible method in order to tame the complexity of the problem resulting, additionally, in an interpretable structure.

## Competing interests

The authors declare that they have no competing interests.

## Authors’ contributions

FES conceived the study. RDMS, ST and FES performed the analysis, interpreted the results and wrote the paper. All authors read and approved the final manuscript.
